# Diagnosis and prognosis of acute hamstring injuries in athletes

**DOI:** 10.1007/s00167-012-2055-x

**Published:** 2012-05-24

**Authors:** Gino M. M. J. Kerkhoffs, Nick van Es, Thijs Wieldraaijer, Inger N. Sierevelt, Jan Ekstrand, C. Niek van Dijk

**Affiliations:** 1 ESSKA Sports Committee, Department of Orthopedic Surgery, Academic Medical Center, 1105 AZ Amsterdam, The Netherlands; 2 Department of Medical and Health Sciences, Linköping University, Linköping, Sweden

**Keywords:** Hamstring strain injury, Sports injury, Physical examination, Imaging

## Abstract

**Purpose:**

Identification of the most relevant diagnostic and prognostic factors of physical examination and imaging of hamstring injuries in (elite) athletes.

**Methods:**

A literature search was conducted in MEDLINE and EMBASE for articles between 1950 and April 2011. A survey was distributed among the members of the European Society of Sports Traumatology, Knee Surgery and Arthroscopy, which focused on physical examination, prognosis, imaging and laboratory tests of hamstring injuries in (elite) athletes.

**Results:**

Medical history, inspection and palpation of the muscle bellies and imaging are most valuable at the initial assessment according to the literature. Experts considered medical history, posture and gait inspection, inspection and palpation of muscle bellies, range of motion tests, manual muscle testing, referred pain tests and imaging to be most important in the initial assessment of hamstring injuries. Magnetic resonance imaging (MRI) is preferred over ultrasonography and should take place within 3 days post-trauma. Important prognostic factors are injury grade, length of the muscle tear on MR images, MRI-negative injuries and trauma mechanism.

**Conclusions:**

Posture and gait inspection, inspection and palpation of muscle bellies, range of motion tests, manual muscle testing and referred pain tests within 2 days post-trauma were identified as the most relevant diagnostic factors.

**Level of evidence:**

Literature review and expert opinion, Level V.

## Introduction

Hamstring injuries are frequent in sports like football [1–17, 21, 27, 46, 59, 60], Australian rules football [43, 52], sprinting [65], American football [20] and rugby [10]. Considering the explosive character of sprinting, it is not surprising that the incidence of injuries (0.87/1,000 h of exposure) is comparable to the incidence in contact sports (0.92–0.96/1,000 h of exposure) [17, 18, 65]. At the top level, professional football team of 25 players can expect about 7 hamstring injuries per season [16]. These injuries frequently cause a significant loss of time from competition and have a high recurrence rate (12–43 %) [11, 17, 21, 24, 27, 30, 35, 39, 46, 52, 56, 58]. Elite football players sustaining a hamstring injury cannot participate in competition for a mean of 14 days [17]. The need for a quick and accurate diagnosis and prognosis of hamstring injuries in elite sports is evident and has been given greater emphasis. The number of games in elite sports has increased and the stakes are higher [63]. However, there is little evidence for the diagnostic and prognostic value of several physical tests [50, 61] and discussion continues on the optimal imaging technique [1, 8, 13, 14, 34, 50, 61]. The objective of this study was to identify the most relevant diagnostic and prognostic aspects of physical examination and additional studies of hamstring injuries in (elite) athletes.

## Materials and methods

### Literature review

A comprehensive literature study was conducted for articles between January 1950 and April 2011 on the diagnosis and prognosis of hamstring injuries. The strategies that were used consisted of searching online databases (MEDLINE and EMBASE) and scanning reference lists. The search terms used in MEDLINE were hamstring* or thigh[MeSH] combined with the MeSH-terms ‘Sprains and Strains/diagnosis’, ‘Muscle, Skeletal/injuries’, ‘Magnetic resonance imaging’ or ‘Ultrasonography’. In EMBASE, the terms ‘hamstring’/exp/mj’ or ‘thigh/exp/mj’ and ‘injury’/exp combined with prognos*, diagnos*, assess*, ultrasonograph*, ultrasound, mri, ‘magnetic resonance imaging’ or imag* were searched for. Articles concerning medical history, physical examination, prognosis and imaging of hamstring injuries were selected.

### Expert opinion

As part of a project of the Sports Committee of the European Society of Sports Traumatology, Knee Surgery and Arthroscopy (ESSKA), ESSKA Members (*n* = 800) were invited by e-mail to participate in an English web-based survey in June 2009. The survey focused on the physical examination, prognosis, imaging and laboratory tests for hamstring injuries in (elite) athletes. The questions were formulated by the authors on the basis of a comprehensive literature review. The survey was a mixture of open questions, multiple choice questions and Likert-scale questions. The five options from which the respondents could choose in the Likert-scale questions were (1) not important, (2) of little importance, (3) moderately important, (4) important and (5) very important. Additional information was provided on the way in which the physical tests were carried out. A pilot survey was distributed among the orthopaedic surgeons of the Academic Medical Center in Amsterdam in order to identify indistinct, irrelevant and missing questions. One week after the first invitation to take part, a reminder was sent by e-mail to the ESSKA members in which they were asked to participate in the survey.

### Statistic analysis

Data were analysed using SPSS version 16.0 (Chicago, USA). Results were mainly presented in a descriptive way as frequencies with corresponding percentages and averages with standard deviations. Likert scales were dichotomised by combining options 1 and 2 and options 3–5 to, respectively, the categories ‘not important’ and ‘important’.

## Results

One hundred and forty ESSKA members (18 % response rate) from 34 countries with 18 (SD 9.6) years of experience completed the questionnaire. The selected articles from the literature search are categorised according to the type of article and the level of evidence in Tables 1, 2.

**Table 1:** Overview of the literature review source articles

Article	Article design (type)	Level of evidence
Allen et al. [1]	Expert opinion/background	V
Arnason et al. [2]	Epidemiological review Retrospective cohort study	II
Arnason et al. [3]	Original article Prospective therapeutic study	II
Askling et al. [4]	Original article Prospective prognostic study	II
Askling et al. [5]	Original article Prognostic case series	II
Askling et al. [6]	Original article Prognostic case series	II
Askling et al. [7]	Original article Prognostic case series	IV
Bencardino et al. [8]	Expert opinion/background	V
Blankerbaker et al. [9]	Expert opinion/background	V
Brooks et al. [10]	Original article Cohort study (prevention)	III
Carling et al. [11]	Epidemiological review Prognostic case series	II
Cohen et al. [12]	Literature review/background	V
Connell et al. [13]	Original article Diagnostic case series	I
Davis [14]	Expert opinion/background	V
Ekstrand et al. [15]	Original article Prospective cohort study	II
Ekstrand et al. [16]	Original article Prospective two-cohort study	II
Ekstrand et al. [17]	Original article Prospective cohort study	II
Ekstrand et al. [18]	Original article Prospective cohort study	II
Ekstrand et al. [19]	Original article Prospective cohort study	II
Elliott et al. [20]	Descriptive epidemiology study Prospective cohort study	II
Engebretsen et al. [21]	Original article Prospective cohort study	II
Fleckenstein et al. [22]	Original article Diagnostic case series (descriptive)	III
Fleckenstein et al. [23]	Expert opinion/background	V
Gibbs et al. [24]	Original article Prospective diagnostic study	I
Gielen et al. [25]	Expert opinion/background Descriptive chapter	V
Guerrero et al. [26]	Original article Prognostic case series	III
Hägglund et al. [27]	Original article Prospective prognostic study	I
Heiderscheit et al. [29]	Expert opinion/background	V
Heiser et al. [30]	Original article Retrospective cohort study	III
Klingele et al. [31]	Original article Retrospective cohort study	III
Kornberg et al. [32]	Original article Therapeutic cohort study	II
Koulouris et al. [33]	Original article Retrospective cohort study	III
Koulouris et al. [34]	Expert opinion/background	V
Koulouris et al. [35]	Original article Prognostic cohort study	III
Lempainen et al. [36]	Original article Retrospective case series	IV
Liemohn et al. [37]	Original article Therapeutic case series	IV
Malliaropoulos et al. [38]	Original article Prognostic cohort study	II
Malliaropoulos et al. [39]	Original article Prognostic cohort study	I
Martínez Amat et al. [40]	Original article Diagnostic cohort study	II
Miñarro et al. [41]	Original article Diagnostic cohort study	IV
Nikolaou et al. [42]	Biomechanical and histological evaluation of muscle	IV
Orchard et al. [43]	Original article Retrospective epidemiologic study	III
Orchard et al. [44]	Expert opinion/background	V
Peetrons [45]	Expert opinion/background	V
Petersen et al. [46]	Original article Prospective cohort study	II
Puranen et al. [47]	Expert opinion/background	V
Sallay et al. [48]	Original article Descriptive case series	III
Sarimo et al. [49]	Original article Retrospective case series	IV
Schneider-Kolsky et al. [50]	Original article Diagnostic cohort study	I
Schneider-Kolsky et al. [51]	Author’s reply	V
Seward et al. [52]	Original article Prospective cohort study	II
Shellock et al. [53]	Expert opinion/background	V
Slavotinek et al. [54]	Original article Prospective RCT	II
Sorichter et al. [55]	Original article Retrospective case–control study	III
Verrall et al. [56]	Original article Prospective prognostic cohort study	II
Verrall et al. [57]	Original article Prospective cohort study	II
Verrall et al. [58]	Original article Prospective cohort study	II
Volpi et al. [59]	Epidemiological review Retrospective cohort study	III
Walden et al. [60]	Original article Prospective cohort study	I
Warren et al. [61]	Original article Prospective observational study	II
Wood et al. [62]	Expert opinion/background	V
Woods et al. [63]	Epidemiological review Prospective cohort study	II
Woods et al. [64]	Epidemiological review Prospective cohort study	II
Yeung et al. [65]	Original article Prospective cohort study	II
Zeren et al. [66]	Original article Diagnostic cohort study	III

Level of evidence is rendered as ranging from I to V in accordance with guidelines from the centre for evidence-based medicine, Oxford, UK

**Table 2:** Summary of the articles used for this literature review and level of evidence

Article type	Number	Level of evidence				
		I	II	III	IV	V
Total	65	6	26	12	6	15
Original article						
Epidemiological review	7		5	2		
Prospective	30	6	21	2	1	
Retrospective	13			8	5	
Literature review	1					1
Expert opinion/background	13					13
Author’s reply	1					1

### Timing of initial physical examination

Traditionally, the clinical assessment of hamstring injuries is based on a thorough medical history and physical examination consisting of posture and gait inspection, inspection and palpation of muscle bellies, ‘range of motion’ tests (ROM tests) and manual muscle testing. In the literature, the initial assessment is often carried out within 12 h to 2 days post-injury [5, 50, 58, 61]. Advantages of an assessment shortly post-injury are the possibility of quick intervention and a more reliable medical history. However, possible signs of swelling and ecchymosis may arise a few days later and consequently may not be noticed at the initial examination [4, 12, 31, 62]. 82 % of the respondents stated that the initial clinical assessment of an (elite) athlete with a suspected hamstring injury should take place within 2 days. This is confirmed in recent work where it is advised to measure active ROM at the end of the second day [38].

### Palpation

Palpation helps identify the site of injury in cranio-caudal direction, because of possible involvement of the free proximal tendon, and determines the injured muscles (lateral: m. biceps femoris, medial: m. semitendinosus and/or m. semimembranosus). Injuries in the m. biceps femoris [13] and more cranially palpated injuries [5] might correlate with a longer rehabilitation interval.

### Flexibility

Flexibility in acute and sub-acute phase was addressed. Flexibility is tested by means of hamstring ROM tests. In case of a hamstring injury, the range of motion of the hip and the knee of the injured leg is significantly decreased compared to the healthy leg [4]. However, the flexibility of the hip in the acute situation is often influenced by pain as a consequence of which the test may be less accurate. Active ROM is decreased in the acute phase of the injury and it is advised to be measured at the end of the second day [38]. Use of the classic ‘sit-and-reach’ test is discouraged in the literature as the testing result is influenced by spinal mobility (i.e. lumbal flexion), leg length, scapular abduction and stretch on the peripheral nerves by dorsiflexion of the ankle joint [37, 41]. Knee active range of motion deficit 48 h after a unilateral posterior thigh muscle injury is an objective and accurate measurement, predicting recovery time in elite athletes [38].

Flexibility tests were pointed out as important by a majority of the respondents. The ‘sit-and-reach’ test was considered to be important despite the above-mentioned negative advice given in the literature. No difference in importance was found between active and passive ROM tests (n.s).

### Strength

Strength of the hamstring muscles can be tested by means of knee flexion and hip extension against resistance. Bilateral comparison is preferred to identify decreased strength of the injured muscle as a result of pain and/or fibre disruption [4]. An alternative to measuring the strength of the hamstring muscles is the ‘take-off-the-shoe’ test (TOST) (or ‘hamstring-drag’ test) in which the patient is asked to take-off the shoe of the injured leg in a standing position with the help of the foot of the healthy leg [66]. Although this test is potentially a valuable addition to the physical examination, the true value should be studied by comparison with magnetic resonance imaging (MRI) and recurrence rates [51].

### Referred pain

An acute disc prolapse at the L5/S1 level may present with hamstring and/or calf pain and limitations in hip joint flexibility, which may mimic a muscle strain. Subtle lumbosacral canal impingement of the L5 nerve root however may in fact also be a common underlying basis for the age-related predisposition towards hamstring injuries [44]. The distinction between real hamstring injuries and ‘back-related’ or ‘neural’ hamstring injuries can be made by the assessment of referred pain with help of an MRI scan [44, 56]. If the distinction remains difficult, imaging-guided cortisone injections to the lumbosacral canal region (L5 nerve root) is a relatively painless and complication-free outpatient procedure with quick recovery that can be used to distinguish the hamstring-spine dilemma.

Pain felt over the area of the ischial tuberosity and radiating down the back of the thigh is often labelled as the ‘hamstring syndrome’ [47].

### Laboratory tests

Traditional biological markers creatine kinase (CK), lactate dehydrogenase (LDH), myoglobin (Mb) and uric acid should not be used for the diagnosis and prognosis of muscle injuries because of their low sensitivity and specificity [55]. More research is needed to determine the real diagnostic and prognostic value of potential markers, such as ‘fast myosin heavy chains’ (fast MHC) [26], ‘skeletal-troponin I’ (sTnI) [55] and ‘alfa-actin’ [40].

Few of the respondents thought that laboratory tests can be of diagnostic or prognostic importance.

The results of the survey are shown in Table 3.

**Table 3:** Importance of different physical tests and additional studies for hamstring injuries in (elite) athletes according to experts

Test	Important (%)	Not important (%)
Palpation to identify the site of injury	97	3
Palpation to identify the injured muscle(s)	95	5
Knee flexion against resistance	94	6
Inspection of the posterior thigh	93	7
Posture and gait inspection	86	14
Hip extension against resistance	86	14
Assessing referred pain	86	14
Active straight leg raise	85	15
Sit-and-reach test	83	17
Passive knee extension	81	19
Active knee extension	80	20
Passive straight leg raise	80	20
Take-off-the-shoe test/hamstring-drag test	79	21
Prognostic laboratory tests	13	87
Diagnostic laboratory tests	4	96

### Imaging

The imaging provides information on the nature and extent of hamstring injuries. The length of a muscle tear on MR images or the cross-sectional area of the muscle tear on ultrasonography (US) is valuable for estimating the convalescent period [13, 19, 24, 35, 50, 52]. 88 % of the respondents in this study use imaging for hamstring injuries in (elite) athletes.

### Imaging technique

MRI and US are the most suitable imaging techniques for depicting hamstring injuries [8, 34]. Connell et al. [13] concluded that MRI and US are equally useful in diagnosing hamstring injuries at baseline. However, MRI is more sensitive for identifying minimal injuries, with less than 5 % of muscle involved: the radiological definition of a grade-I muscle injury [13, 33, 45]. When imaging is indicated, MRI is used in 40–77 % of cases, both MRI and US in 7–40 % and US only in 20–53 % of cases [10, 17, 19, 64]. The most important advantages and disadvantages of both imaging techniques are presented in Table 4.

**Table 4:** Advantages and disadvantages of MRI and US as imaging technique for hamstring injuries

Qualities	MRI	US
Low costs [13]	−	++
Independence of the quality and experience of the physician [13]	++	−
Ease of use	±	++
Ease of use for prognosis [13]	++	+
Sensitivity for low-grade injuries [13, 33]	+	±
Diagnosis of avulsion fractures [33]	+	±
Reproducibility	++	±
Dynamic assessment	−	++
Availability	±	++
Evaluation of superficial structures [33]	+	++
Evaluation of deep structures	++	±
Correct reflection of the extent of the injury [13]	++	±
Assessment time	±	++
Follow-up imaging [13]	++	+

++ = much applicable, + = applicable, ± = less applicable, − = not applicable

### Time of imaging

There is currently no consensus in the literature on the ideal moment of imaging of hamstring injuries. Ekstrand et al. [19] are in favour of MRI within 24–48 h post-trauma, whereas Gielen et al. [25] argue that a hamstring injury can only be correctly graded 48–72 h post-trauma. Signs of muscle injury on MR images are mainly seen on fat-suppressed T2 images or ‘short-tau inversion recovery’ images (STIR) and are most evident at 24 h to 5 days post-trauma [22, 23, 53]. In prospective studies, MRI is often used out 2–5 days post-trauma [13, 35, 58]. Since the amount of oedema is histologically maximal after 24 h and already decreases after 48 h [42], imaging 1–2 days post-trauma seems to be the best moment.

The respondents prefer imaging within 3 days post-trauma for MRI (66 %) and for US (79 %).

### Follow-up imaging

MRI is more sensitive for follow-up imaging than US [13]. Follow-up imaging is useful in the case of complications and in order to follow the progression of the rehabilitation and consequently to support the decision for sports resumption for (elite) athletes [9]. After 6 weeks in 34–94 % of all cases, signs of hamstring injury are still noticeable on MR images [5]. The ideal moment of follow-up imaging differs in every single case and is therefore difficult to generalise.

66 % of the respondents use follow-up imaging in the case of persisting bad rehabilitation and 61 % to assess the progression of the rehabilitation. In total, 91 % of the respondents use follow-up imaging for hamstring injuries in (elite) athletes.

### Prognostic factors

An accurate prognosis can be obtained on the basis of a thorough clinical assessment [50].

Different classification systems are provided in the literature. A clinical classification system resulting from the treatment for 165 elite track and field athletes with acute, first-time unilateral hamstring muscle strains was proposed in 2010. Strains were classified into 4 grades (I, II, III and IV) based on knee active range of motion deficit at 48 h [38].

Imaging is a valuable addition. There is the classic radiological grading system of a hamstring injury with grade I (minimal muscle damage with <5 % of muscle length involved) or II (partial rupture with 5–50 % of muscle length involved) on MRI or US to correspond with the rehabilitation period [13, 19, 24, 50, 52]. Grade III hamstring injuries (complete rupture or avulsion fracture) are serious injuries resulting in a convalescent period of 3 months up to 1.5 years, often requiring surgery [31, 36, 48, 49]. In 2002, an additional grading systems was introduced, specifically for US, grade 0 (normal US appearance), grade 1 (subtle ultrasound findings), grade 2 and grade 3 injuries (partial and complete muscle tears) [45]. In general, the grading should be done by a team consisting of an orthopaedic surgeon and/or a sports medicine physician and a radiologist.

A substantial part of supposed hamstring injuries are negative on MRI (14–45 %) [24, 50, 56, 57]. In these cases, the symptoms are probably not the result of muscle fibre disruption, but are caused by referred pain (e.g. from the lumbar spine) or abnormal neural tension [32, 44, 47]).

It has been described that hamstring injuries that result from excessive slow-speed stretching require a much longer convalescent period compared to hamstring injuries sustained during high-speed running. In the former type of injury, the m. semimembranosus and the free proximal tendon are often involved, resulting in a rehabilitation period of 31–50 weeks. [6, 7].

Athletes sustaining a recurrent hamstring injury have a longer convalescent period compared to a first-time hamstring injury [10, 17, 18, 35, 39]. The time to return to sports after re-injury was—depending on the injury grade—on average 1.9–11 days longer [10, 17, 35, 39]. Over 50 % of re-injuries occur within 1 month after the initial injury [10]. This emphasises the risk of an early return to sport after a hamstring injury.

The factors mentioned in the literature associated with a longer rehabilitation period are compared with the expert opinion in Table 5.

**Table 5:** Prognostic factors during the injury period associated with a longer rehabilitation period for hamstring injuries in (elite) athletes

Factors associated with a longer rehabilitation period	Literature	Expert opinion
Complete rupture or avulsion fracture [12, 31, 48, 62]	++	++
Greater length of muscle tear on MR images or larger cross-sectional area of muscle tear on ultrasound images [13, 24, 50, 54]	++	++
MRI-positive hamstring injury [13, 24, 57]	++	+
Recurrent hamstring injury [10, 17, 18, 35, 39]	+	++
Persisting pain/restriction at ROM tests, strength tests and sport exercises	+	++
Injury resulting from excessive slow-speed stretching [4]	+	+
Persisting signs of injury on follow-up imaging [5]	+	+
Injury to the m. biceps femoris [13]	±	+
Sports type [5]	±	+
More cranially palpated injury [5]	±	+
Large and deep haematoma	−	++
Hamstring injury involving the free proximal tendon [6]	+	−
Higher subjective pain score at the time of injury on a Visual Analogue Scale (VAS) [57]	+	−
Being unable to walk pain-free within 24 h of injury [61]	+	−
Long period until initial treatment	−	+
Low quality of the rehabilitation programme and minimal willingness of the patient to rehabilitate	−	+

++ = multiple randomised controlled trials (RCT) (strong evidence), + = one RCT (moderate evidence), ± = contradiction in the literature, − = no evidence

## Discussion

In this study, we identified posture and gait inspection, inspection of the muscle bellies, location and extent of the muscle tear, flexibility, strength of the hamstrings and assessing referred pain within 2 days as most relevant diagnostic factors of the physical examination. This is in line with the evidence from recent literature. Consensus between literature and experts was also observed for the use of MRI to identify injuries with a longer rehabilitation time: (1) greater length of muscle tear on MR images, (2) MRI-positive injuries and (3) persistent signs on follow-up injuries.

Although both MRI and US techniques are used, most experts prefer MRI to US. MRI is more sensitive than US [13, 33], easier to use for prognostic purposes [13, 52] and less operator-dependent [33]. Consequently, MRI should be given the preference over US as imaging technique at the initial assessment. Although it has to be stated that US remains valuable in many cases because of its low costs, availability, dynamic character and secondly, that US is indeed still quite operator-dependent.

Consensus between literature and experts was also found for the interpretation that apart from the MRI results, trauma mechanism and injury grade are important prognostic factors and that laboratory tests are of minimal diagnostic and prognostic value.

There was no consensus between literature and experts on the interpretation of a large and deep haematoma: experts interpret a large and deep haematoma as an important factor associated with a longer rehabilitation period; however, literature does not provide the evidence for this. This is also observed for a longer period to initial treatment and low quality of the rehabilitation programme. All three above-mentioned factors seem logical; however, literature does not support nor deny the prognostic value of these factors. There was also no consensus on the value of higher VAS score [58] nor being able to walk pain-free within 24 h of injury [61], experts do not (yet) seem to link these factors to a longer rehabilitation period. Reason for this difference between literature and experts could be the subjective nature of these findings or simply that these findings are not widely known or accepted by other experts yet.

There are obvious limitations of this research. Since many prospective studies evaluated hamstring injuries in only one type of sports, there is a selection bias [2, 3, 10, 11, 15–17, 20, 21, 27, 43, 46, 52, 59, 60, 64, 65]. The question rises whether the conclusions of these studies can be extrapolated to other sports types. In our survey, experts were not asked to specify for the sports types with which they deal in their daily practice. Caution is therefore recommended when adopting the results of this study.

Also, there is the limited value of a questionnaire with low response rate, again a selection bias [28]; however, we feel that the research benefits from the information provided by the selection of ESSKA members with interest in muscle injuries that answered the questionnaire.

We feel that there is a definite need for further research in this field. First, all prognostic factors identified in this review should be validated in a prospective cohort and even better in different cohorts of active sports participants, so the difference between the different sport types can also be monitored. Second, the anatomy of the hamstring (injuries) can be re-evaluated to see whether we can identify important prognostic factors on a basic level. Third, imaging provides numerous keystones to improve the understanding of the extent of the hamstring injury and to link this to an accurate prognosis: ideal moment of initial assessment, use of follow-up imaging in decision-making, US versus MRI, optimalisation of MRI modalities.

**Fig. 1:**
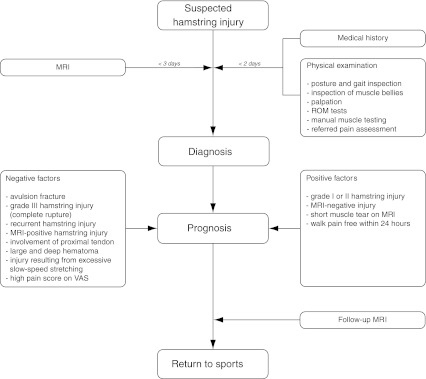
Guideline for diagnosing hamstring injuries and estimating the convalescent period in elite athletes

With this combination of best evidence from the literature and experts from the field, the most relevant and explicit diagnostic and prognostic factors of physical examination, imaging and additional studies of hamstring injuries in (elite) athletes were identified and an assessment protocol for hamstring injuries in (elite) athletes (Fig. 1) was proposed. In this way, it was attempted to provide a guideline for diagnosing hamstring injuries and estimating the convalescent period in (elite) athletes.

## Conclusion

Physical examination of an athlete with suspected acute hamstring injury should take place within 2 days post-trauma and consists of posture and gait inspection, location and extent of the muscle tear, flexibility and strength of the hamstrings and assessing referred pain.

MRI as imaging technique for acute hamstring injuries in elite athletes is preferred over ultrasound by both the experts and recent literature mainly based on its greater sensitivity for minor injuries and the ease of use for an accurate prognosis.

Important prognostic factors related to a longer rehabilitation period are MRI-positive muscle tears, larger extent of the muscle tear as seen on MRI, recurrent hamstring injury and injury mechanism.
